# Uridine Improves Locomotor Activity and Sciatic Nerve Integrity in a Mouse Model of Diabetes Mellitus

**DOI:** 10.3390/biom16050750

**Published:** 2026-05-20

**Authors:** Anca-Maria Țucă, Smaranda Ioana Mitran, Emilia Burada, Alexandra Nicoleta Preda, Alexandra Oltea Dan, Elena-Anca Târtea, Andrei Greșiță, Răzvan-Cosmin Pană, Diana-Ruxandra Hădăreanu, Veronica Sfredel, Georgică Târtea

**Affiliations:** 1 Experimental Research Centre for Normal and Pathological Aging, University of Medicine and Pharmacy of Craiova, 2 Petru Rares Street, 200349 Craiova, Romania; anca.tuca@umfcv.ro (A.-M.Ț.); smaranda.mitran@umfcv.ro (S.I.M.); emilia.burada@umfcv.ro (E.B.); departament2.medicina@umfcv.ro (A.N.P.); alexandra.dan@umfcv.ro (A.O.D.); andrei.gresita@umfcv.ro (A.G.); veronica.sfredel@umfcv.ro (V.S.); georgica.tartea@umfcv.ro (G.T.); 2 Department of Neurology, University of Medicine and Pharmacy of Craiova, 2 Petru Rares St., 200349 Craiova, Romania; 3 Department of Gynecology, University of Medicine and Pharmacy of Craiova, 2 Petru Rares St., 200349 Craiova, Romania; razvan.pana@umfcv.ro; 4 Department of Cardiology, University of Medicine and Pharmacy of Craiova, 2 Petru Rares St., 200349 Craiova, Romania

**Keywords:** uridine, locomotor activity, neuroprotection, diabetic peripheral neuropathy

## Abstract

Diabetic peripheral neuropathy is an important cause of functional disability, and current therapies have limited ability to halt its progression. Uridine, a pyrimidine nucleoside essential for the synthesis of membrane phospholipids and neuronal metabolism, appears to be a potential neuroprotective agent, but its impact on motor behavior and peripheral nerve integrity in diabetes remains insufficiently investigated. Our study investigated the effects of chronic uridine supplementation on locomotor performance, neuromuscular electrophysiological manifestations, and morphological changes in the sciatic nerve in a murine model of streptozotocin-induced diabetes. We used male C57BL/6 mice (*n* = 8/group) that were assigned to three groups: sham (no diabetes), diabetic (streptozotocin-induced, diabetes mellitus, DM+), and diabetic treated with uridine (DM+U). We observed that uridine did not alter the metabolic status, as the HbA1c values remained comparable between diabetic groups (9.93 ± 0.57% DM+ vs. 9.71 ± 0.55% DM+U; *p* = 0.72), suggesting effects independent of glycemic control. The open field test revealed that diabetic mice showed a marked reduction in spontaneous locomotion, while uridine-treated mice maintained a significantly higher level of activity (longer total distance traveled 3761.7 ± 789.1 cm vs. 2477.5 ± 1017.6 cm in DM+; *p* = 0.023). Electrophysiological evaluation revealed near-normal sciatic nerve function in DM+U mice, including higher compound motor action potential (CMAP) amplitudes (10.21 ± 0.64 mV vs. 5.75 ± 0.72 mV; *p* < 0.0001) and reduced F-wave latency (6.35 ± 0.45 ms vs. 7.29 ± 0.31 ms; *p* < 0.0001). Histological and immunohistochemical analyses (PGP 9.5) further confirmed reduced nerve degeneration in DM+U mice. Our data suggest that chronic uridine administration may confer both functional and structural neuroprotection in diabetic neuropathy, even in the absence of improved glycemic control.

## 1. Introduction

Globally, diabetes mellitus is one of the most important public health problems. The 10th Edition of the Diabetes Atlas of the International Diabetes Federation (IDF) estimated that, in 2021, approximately 537 million adults (10.5% of the population aged 20–79 years) were living with diabetes, a number that will reach 783 million by 2045 [1]. More recently, the 11th edition (2025) indicated that 11.1% of adults, equivalent to approximately 590 million people, have diabetes, with 853 million cases expected by 2050 [2]. The global burden of the disease is amplified by chronic micro- and macrovascular complications, of which diabetic peripheral neuropathy (DPN) occupies a central place.

DPN, especially distal symmetric polyneuropathy (DSPN), affects up to 50% of patients with diabetes [3]. Recent studies show that 10–15% of patients with type 2 diabetes show signs of DSPN at the time of diagnosis, with the prevalence exceeding 50% after a diabetes duration of more than 10 years [4,5,6,7]. Clinically, patients with DPN present with neuropathic pain, sensory loss, gait disturbance, increased risk of falls, foot ulcers and amputations, and a marked deterioration in the quality of life [5,7]. Recent analyses of the global burden of DPN highlight the major impact of this complication on mortality, disability, and costs of care, underlining the urgent need for therapeutic strategies for this disease [5,8,9]. The pathogenesis of DPN is complex and multifactorial. Recent studies show that chronic hyperglycemia, dyslipidemia, and insulin resistance activate a cascade of metabolic pathways—including the polyol pathway, the formation of advanced glycation end products (AGEs), protein kinase C activation, oxidative stress, and mitochondrial dysfunction—that, together with microangiopathy and neuroinflammation, contribute to axonal and Schwann cell injury [5,10,11]. There is also increasing evidence that inflammation plays a critical role in the initiation and progression of DPN, both through systemic mechanisms and through local pathways in the peripheral nerve [12,13,14,15,16,17,18]. Despite advances in understanding these mechanisms, the therapeutic options remain limited: intensive glycemic control can delay or reduce the risk of neuropathy in type 1 diabetes, but it has a more modest effect in type 2 diabetes, and current treatment focuses mainly on relieving painful symptoms without halting disease progression [3,5]. Experimental models are essential for exploring pathogenetic mechanisms and evaluating new neuroprotective strategies [5].

Uridine has emerged as a promising neuroprotective molecule, due to its involvement in membrane phospholipid biosynthesis, mitochondrial energy metabolism, RNA synthesis, and the maintenance of neuronal structural integrity [11,12,13,14,15,16,17,18,19,20,21,22,23,24,25]. Clinical and experimental studies suggest that uridine may exert beneficial effects in diabetic and other peripheral neuropathies, although the available evidence remains limited and heterogeneous.

Clinical studies have reported that prolonged oral administration of uridine in patients with diabetic neuropathy is associated with improved nerve conduction velocity, enhanced nerve fiber regeneration, and better myelin morphology [11,12]. Gallai et al. also demonstrated significant neurophysiological improvements in peripheral nerves following uridine treatment [12]. In addition, combinations containing uridine monophosphate and B vitamins have shown beneficial effects in painful peripheral neuropathies, including reductions in neuropathic pain intensity, the number of painful areas, and analgesic consumption, suggesting possible effects on myelin repair and nerve regeneration [13].

At the preclinical level, combinations of cytidine, uridine, and gabapentin demonstrated analgesic and neuromodulatory effects in experimental neuropathy models, including STZ-induced diabetic neuropathy, partly through the modulation of p-CREB expression [14,15,16,17,18,19,20]. These findings support the potential neuroprotective role of uridine at both central and peripheral levels. However, most available studies have focused primarily on sensory symptoms and nerve conduction parameters, while relatively little is known about the effects of uridine on global locomotor activity and sciatic nerve functional integrity in experimental diabetes. Moreover, these interactions remain insufficiently explored in conditions where glycemic control is not significantly improved. Since diabetic peripheral neuropathy may progress despite apparently adequate glycemic control, therapies with direct neuroprotective and neuromodulatory properties remain of considerable interest [5]. The aim of our study was to investigate the effects of chronic uridine administration on locomotor activity, electromyographic parameters, and sciatic nerve integrity in streptozotocin-induced diabetic mice, in the absence of a significant reduction in blood glucose and in the presence of a comparable metabolic state.

## 2. Materials and Methods

### 2.1. Laboratory Animals and Experimental Conditions

#### 2.1.1. Ethical Approval

We conducted a prospective experimental study, in accordance with the institutional, national, and international regulations regarding the use of laboratory animals. The protocol was approved by the Ethics Committee of the University of Medicine and Pharmacy (UMP) of Craiova (approval number 36/20.01.2023). The UMP Craiova facility operates under FELASA accreditation, and all procedures complied with the European Council directives on the protection of animals used for scientific purposes (86/609/EEC). Morphopathological analyses were performed within the Research Center for Microscopic Morphology and Immunological Studies of UMP Craiova.

#### 2.1.2. Animals and Maintenance Conditions

For this study, we used 24 male C57BL/6 mice, aged 8–10 weeks and weighing between 19 and 27 g. This model faithfully reproduces the major pathogenic mechanisms of diabetic neuropathy (chronic hyperglycemia, oxidative stress, mitochondrial dysfunction, axonal damage), which are widely validated in the preclinical literature and relevant for studying the mechanisms involved in human DPN [15]. The animals were maintained in an SPF (specific-pathogen-free) environment, under temperature-controlled conditions (22 ± 1 °C) and a 12/12 h light/dark cycle, with ad libitum access to food and water. Before all procedures, the mice were removed from the maintenance unit and allowed to acclimatize.

#### 2.1.3. Induction of Diabetes Mellitus and Metabolic Monitoring

Diabetes was induced by administering a single dose of 150 mg/kg streptozotocin, injected intraperitoneally, as in our previous studies [23]. Diabetes was confirmed according to the ADA diagnostic criteria, defined as persistent fasting blood glucose levels > 126 mg/dL following STZ administration [1]. We measured the blood glucose weekly, starting from the moment of inclusion of the animals in the study and, subsequently, after the induction of diabetes, using blood collected from the caudal vein and a Contour Plus One manual glucometer (Ascensia Diabetes Care, Basel, Switzerland). For the dosage of glycosylated hemoglobin (HbA1c), we used the OnCall MultiPro HbA1c analyzer (ACON Laboratories, San Diego, CA, USA).

#### 2.1.4. Experimental Groups

The 24 mice were randomly divided into three groups (*n* = 8/group):

- Sham group (DM−): non-diabetic mice, without streptozotocin, without uridine, who received only 0.9% saline solution intraperitoneally. In this group of animals, we evaluated clinical tests (weight, blood glucose, HbA1c), behavioral tests (open field test), and electromyographic tests, as well as tests to morphologically evaluate the sciatic nerve in the absence of diabetes.
- Control group (DM+): STZ-induced diabetic mice, without further treatment. Clinical, functional, electrophysiological, and structural parameters of the sciatic nerve were analyzed.
- Treatment group (DM+Uridine): diabetic mice who received uridine 40 mg/kg/day via gastric gavage, for 12 weeks, starting after biochemical confirmation of hyperglycemia. Uridine (product code U1752) was purchased from Sigma-Aldrich and freshly prepared before administration, according to the experimental protocol. Compared with previously reported acute high-dose paradigms, we selected a lower chronic dose suitable for prolonged administration over 12 weeks, while also aiming to minimize the potential metabolic adverse effects associated with high-dose uridine administration, including the development of insulin resistance reported in some studies [22,23]. This group of animals was also subjected to the previously mentioned tests.

The sample size (*n* = 8 animals/group) was determined based on previous experimental studies investigating diabetic neuropathy and neuroprotective interventions in STZ-induced diabetic mice, considering the expected effect sizes, the variability of electrophysiological parameters, and ethical principles aimed at minimizing animal use [23]. Animals were randomly allocated into the experimental groups using simple randomization generated by a computerized random number generator.

### 2.2. Behavioral Evaluation. Open Field Test

The conditions for this test were standardized, with low lighting, a temperature of 22 ± 1 °C, and minimal ambient noise. Mice were acclimatized to the test room for at least 30 min before the start of the test.

#### 2.2.1. Test Procedure

To perform this test, we used a light-colored Plexiglas arena (50 × 33 × 15 cm), virtually divided into a central and a peripheral area. Each animal was individually placed in the center and monitored for 10 min using a video camera mounted above the arena. Between tests, we cleaned this arena with 70% ethanol to eliminate residual olfactory signals and to not influence the behavior of the other animals.

#### 2.2.2. Parameters Analyzed

For this test, we used EthoVision XT 14 software (Noldus, Wageningen, The Netherlands) to quantify the locomotor and behavioral parameters, including:

- Average movement speed (cm/s);
- Total distance traveled (cm);
- Time spent in the central and peripheral areas (s);
- Duration of locomotion and duration of immobilization (s).

### 2.3. Tissue Collection and Histological Analysis

#### 2.3.1. Tissue Sample Preparation

The object of tissue analysis was the sciatic nerves. To collect these specimens, we deeply anesthetized the mice for euthanasia. Subsequently, the sciatic nerves were excised and fixed in 4% formalin for 24–48 h. Next, these specimens were washed for 24 h to remove the fixative, and then, they were embedded in paraffin. From the fixed samples, we formed 4 μm serial sections using the HM355S automated microtome, which were successively transferred to cold and then warm water baths (40 °C), mounted on poly-L-lysine-treated slides, and incubated for 24 h at 60 °C.

#### 2.3.2. Staining and Immunohistochemistry Procedures

The initial staining was performed using the hematoxylin–eosin technique. To assess the integrity of the peripheral nervous system, we used a primary antibody anti-PGP 9.5 (a rabbit recombinant Anti-PGP 9.5 antibody [EPR4118], Neuronal Marker, ab108986, Abcam, 1:200). The sections were subsequently incubated with a secondary antibody conjugated with horseradish peroxidase (HRP), and visualization was performed using a 3,3′-diaminobenzidine (DAB) substrate, followed by counterstaining with hematoxylin.

#### 2.3.3. Microscopic Analysis and Imaging Quantification

Initially, sections were analyzed using a Nikon 55i microscope (Nikon, Tokyo, Japan) equipped with a 5 MP camera, with Image ProPlus AMS 9 software. Subsequently, all slides were completely scanned using the MoticEasyScan Pro 6 system (20× objective), and the images were digitized for analysis. The total section area and integrated optical density (IOD) were calculated, and the values were reported relative to the total area of the analyzed nerve.

### 2.4. Electrophysiological Monitoring

#### 2.4.1. Recording System and Procedural Conditions

The electrophysiological properties of the sciatic nerve were evaluated using the Neuro MEB-4 electromyography system (Neurosoft Ltd., Ivanovo, Russia), software version 4.0.

Anesthesia was performed with a ketamine–xylazine cocktail, and its depth was verified by the pinch reflex [19,20,21,22,23].

Bepanthen ophthalmic ointment (Bayer, Leverkusen, Germany) was applied to protect the cornea. Recordings were performed using four monopolar needle-type electrodes.

We used the following technical parameters:

- Frequency band: 20 Hz–10 kHz;
- Sweep speed: 1 ms/division;
- Sensitivity: 5 mV/division.

#### 2.4.2. Quantified Electrophysiological Parameters

For each animal, we applied three electrical stimuli (duration 0.2 ms), with intensities of 5–6 mA, which were increased to 7 mA in isolated cases to obtain the maximum CMAP. Recordings were made at the level of the gastrocnemius muscle.

The following parameters were analyzed:

- CMAP: amplitude (indicative of axonal integrity), duration (reflects demyelination), and latency to peak;
- F-wave: latency, amplitude, and duration, useful for detecting proximal damage in diabetic polyneuropathy.

The final values analyzed were the mean of the maximum values obtained from the three stimulations. Basal values were recorded before diabetes induction.

### 2.5. Statistical Analysis

Data are expressed as the mean ± standard deviation. Raw information was collected in Microsoft Excel and subsequently exported to GraphPad Prism v11 software (La Jolla, CA, USA) for statistical analysis.

Differences between groups were evaluated using the ANOVA test, with the statistical significance level set at *p* < 0.05. Data normality was assessed using the Shapiro–Wilk test prior to parametric statistical analyses. Comparisons between groups were performed using one-way ANOVA or two-way repeated measures ANOVA, followed by Tukey’s post hoc test where appropriate. Exact F values, degrees of freedom (df), and *p*-values are reported throughout this article. Effect sizes were estimated using partial eta squared (partial η^2^). We also used the following symbols to highlight the strength of statistical significance: * *p* < 0.05, ** *p* < 0.01, *** *p* < 0.001, **** *p* < 0.0001.

The study was conducted and reported in compliance with the ARRIVE 2.0 guidelines, with detailed descriptions provided for the experimental design, randomization procedures, housing conditions, and statistical analysis to ensure transparency and reproducibility [24]. Histological analysis and data interpretation were performed by investigators blinded to group allocation. No animals or data points were excluded from the analysis.

## 3. Results

### 3.1. Evaluation of Clinicopathological Parameters

#### 3.1.1. Evolution of Plasma Glycemia

**Figure 1:**
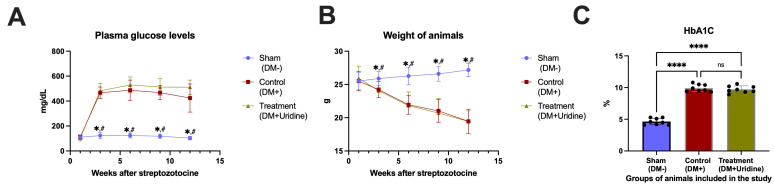
Evolution of metabolic parameters in the three experimental groups. (**A**) Plasma glucose measured before STZ administration and then 3, 6, 9, and 12 weeks after STZ administration. (**B**) Evolution of the body weight for the animals included in the study. (**C**) HbA1c values at the end of the study. * *p* < 0.05 vs. Control (DM+), # *p* < 0.05 vs. Treatment (DM+Uridine), **** *p* < 0.0000. ns, not significant.

The blood glucose levels in the sham group (DM−) remained stable, ranging from 110.0 ± 20.35 mg/dL before the start of the experiment to 103.1 ± 14.62 mg/dL 12 weeks after the start of the experiment. In contrast, untreated diabetic mice (Control, DM+) showed severe and persistent hyperglycemia, with values increasing from 111.9 ± 16.17 mg/dL before streptozotocin (STZ) administration to 424.0 ± 113.32 mg/dL 12 weeks after STZ. Uridine administration did not alter the blood glucose levels compared to the DM+ group, with the values remaining at similar levels (e.g., 105.1 ± 24.83 mg/dL at the start of the experiment and 511.5 ± 56.95 mg/dL 12 weeks after STZ). Two-way repeated measures ANOVA revealed significant effects of time (F(2.554, 53.63) = 99.35, *p* < 0.0001), experimental group (F(2, 21) = 601.3, *p* < 0.0001), and time × group interaction (F(5.108, 53.63) = 24.15, *p* < 0.0001) on the plasma glucose levels. Statistical analysis confirmed significant differences between the sham group and the two diabetes mellitus groups starting from week 3 (e.g., at week 3: DM− vs. DM+ *p* < 0.0001; DM− vs. DM+Uridine *p* < 0.0001), while the differences between DM+ and DM+Uridine did not reach statistical significance at any time point in the study (*p* > 0.05, Figure 1A). Partial eta squared analysis demonstrated very large effect sizes for plasma glucose levels, particularly for the experimental group (partial η^2^ = 0.983), time (partial η^2^ = 0.826), and time × group interaction (partial η^2^ = 0.697), indicating a strong influence of diabetes induction on the glycemic status.

#### 3.1.2. Animal Body Weight

The sham (DM−) group showed a progressive increase in body weight, from 25.52 ± 1.32 g at inclusion in the study to 27.17 ± 1.04 g at 12 weeks after STZ administration. In contrast, diabetic mice in the control group showed a gradual and pronounced decrease in weight, reaching 19.46 ± 1.82 g at the end of the experiment. Uridine administration did not prevent the weight loss specific to diabetes, with the weight of animals in the DM+Uridine group at 12 weeks at 19.44 ± 1.59 g, comparable to that in the DM+ group. Two-way repeated measures ANOVA revealed significant effects of time (F(3.041, 63.87) = 47.74, *p* < 0.0001), experimental group (F(2, 21) = 25.45, *p* < 0.0001), and time × group interaction (F(6.082, 63.87) = 24.41, *p* < 0.0001) on body weight evolution. The differences between the sham group and the two diabetic groups became significant starting with week 6 (e.g., at 6 weeks: DM− vs. DM+ *p* < 0.0001; DM− vs. DM+Uridine, *p* = 0.0007). As with glycemia, no significant differences were identified between the DM+ and DM+Uridine groups (Figure 1B). The body weight evolution was associated with large effect sizes for time (partial η^2^ = 0.694), experimental group (partial η^2^ = 0.708), and time × group interaction (partial η^2^ = 0.699), supporting a marked effect of diabetes progression on body weight dynamics.

#### 3.1.3. Glycosylated Hemoglobin (HbA1c)

The HbA1c values were significantly higher in the diabetic groups compared to the sham group (DM−), confirming the chronic hyperglycemic status. The mean HbA1c value was 4.65 ± 0.52% in the sham group, 9.93 ± 0.57% in the control group (DM+), and 9.71 ± 0.55% in the DM+Uridine group. One-way ANOVA confirmed a significant overall group effect (F(2, 21) = 237.6, *p* < 0.0001). There was also a very large effect size for HbA1c differences between groups (partial η^2^ = 0.958), confirming the impact of diabetes on long-term glycemic control.

ANOVA analysis revealed highly significant differences between the sham group and the diabetic groups (DM− vs. DM+ *p* < 0.0001; DM− vs. DM+Uridine *p* < 0.0001), but no significant differences were observed between DM+ and DM+Uridine (*p* = 0.7217). These results confirm that uridine treatment does not influence the HbA1c values, which supports our observation that the potential mechanisms of neuropathy improvement are independent of glycemic control (Figure 1C).

### 3.2. Assessment of Locomotor and Exploratory Behavior

**Figure 2:**
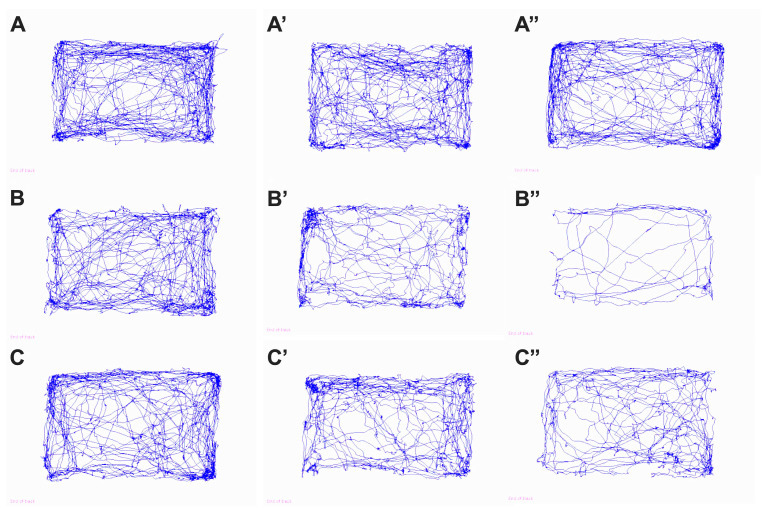
Movement maps in the open field test in sham (**A**), untreated diabetic (**B**), and uridine-treated diabetic (**C**) mice. Images (**A**–**C**) represent the movement maps before diabetes induction. (**A′**–**C′**) illustrate the movement maps 8 weeks after diabetes induction. (**A″**–**C″**) reflect the situation at 12 weeks.

**Figure 3:**
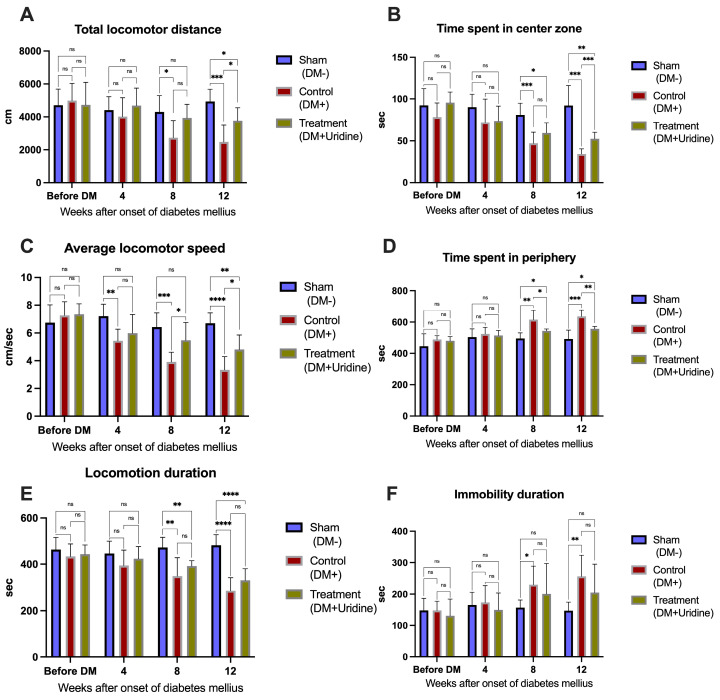
Quantitative analysis of locomotor activity and exploratory behavior in the open field test at 0, 4, 8, and 12 weeks after diabetes induction. (**A**) Total distance traveled; (**B**) time spent in the central zone; (**C**) average speed of movement; (**D**) time spent in the peripheral zone; (**E**) duration of locomotion; (**F**) duration of immobilization. Data represent the mean ± SD. Significance levels: * *p* < 0.05, ** *p* < 0.01, *** *p* < 0.001, **** *p* < 0.0001. ns, not significant.

Qualitative analysis of trajectories in the open field test (Figure 2) highlights the evolution of locomotor and exploratory behavior in the studied mice. Quantitative data are presented in Figure 3. In the prediabetic stage, before STZ administration, the movement maps are homogeneous between the three groups, with dense and well-distributed trajectories throughout the arena, including the central area, reflecting a comparable level of motor activity. This observation is consistent with the statistical analysis, which indicates no significant differences between the groups at baseline for any of the locomotor parameters (*p* > 0.05), confirming the absence of pre-existing behavioral variations.

At 8 weeks after diabetes induction, the hyperglycemic changes become clearly visible in the control (DM+) group. The trajectories of these mice are considerably reduced in density, with movement being predominantly limited to the periphery of the arena, with avoidance of the central area—a classic indicator of increased anxiety-like behavior and reduced exploratory activity. At this stage, the reduction in locomotor performance is also reflected in the quantitative data: at 8 weeks, the DM+ group covers a significantly shorter distance than the sham group (difference of 1567 cm, *p* = 0.0205), and the average movement speed is also reduced (difference of 2.508 cm/s compared to sham, *p* = 0.0002). In contrast, at 8 weeks, diabetic mice treated with uridine (DM+Uridine) present a movement map closer to that of the sham group, maintaining repeated crossings of the arena and use of the central area. These observations are supported by the quantitative data, which show that the distance traveled and locomotor speed in the treated group are superior to those in the DM+ group, with no significant differences compared to the sham group at 8 weeks, suggesting an early protective effect of uridine on locomotor function (Figure 3A,C).

At 12 weeks, the contrast between the groups becomes much more pronounced. In the DM+ group, the trajectory maps (Figure 2B″) are deeply rarefied, with slow and predominantly peripheral movements, reflecting the severe reduction in mobility and the accentuation of immobilization, a phenomenon characteristic of advanced diabetic neuropathy. This behavioral degradation is quantitatively confirmed: the total distance traveled decreases to approximately 2477.5 ± 1017.6 cm, significantly lower than in the sham group (4929.4 ± 751.39 cm, *p* = 0.0003, Figure 3A), and the average movement speed is markedly reduced (3.34 cm/s vs. 6.69 cm/s, *p* < 0.0001, Figure 3C). At the same time, the duration of immobilization increases significantly in the diabetic group (*p* = 0.0049, Figure 3B). Immobilization in the peripheral area also increases, indicating an increased level of anxiety (*p* = 0.0002 vs. sham, Figure 3D). In contrast, mice treated with uridine (Figure 2C″) maintain significantly better motility, with dense trajectories distributed over a large part of the arena, reflecting a visibly higher level of exploratory activity compared to DM+. This visual impression is confirmed by the quantitative analysis: the total distance traveled reaches approximately 3761.71 ± 789.09 cm, significantly higher compared to DM+ (*p* = 0.0230, Figure 3A). In addition, uridine significantly improves the average movement speed (*p* = 0.0033, Figure 3C) and reduces the time spent in the peripheral zone (*p* = 0.0012, Figure 3D), suggesting an improvement in the anxiety profile. The treated mice also spend more time in the central zone than untreated ones (*p* = 0.0005), a further indicator of the restoration of exploratory behavior.

Although the duration of immobilization is shorter in DM+Uridine compared to DM+, this difference does not reach statistical significance at 12 weeks (*p* > 0.05); however, the general trend indicates a favorable effect on the global motor profile.

Two-way repeated measures ANOVA demonstrated significant effects of time, experimental group, and time × group interaction for several locomotor and behavioral parameters assessed in the open field test. Significant effects were observed for locomotion duration (time: F(2.815, 59.12) = 28.20, *p* < 0.0001; group: F(2, 21) = 10.18, *p* = 0.0008; interaction: F(5.631, 59.12) = 13.44, *p* < 0.0001), time spent in the peripheral zone (time: F(2.381, 50.00) = 31.31, *p* < 0.0001; group: F(2, 21) = 13.98, *p* = 0.0001; interaction: F(4.762, 50.00) = 5.714, *p* = 0.0004), time spent in the center zone (time: F(2.565, 53.87) = 28.59, *p* < 0.0001; group: F(2, 21) = 12.12, *p* = 0.0003; interaction: F(5.131, 53.87) = 6.463, *p* < 0.0001), and average locomotor speed (time: F(2.176, 45.69) = 40.20, *p* < 0.0001; group: F(2, 21) = 10.88, *p* = 0.0006; interaction: F(4.352, 45.69) = 10.26, *p* < 0.0001). For the total locomotor distance, significant effects of time (F(2.247, 47.19) = 33.09, *p* < 0.0001) and time × group interaction (F(4.495, 47.19) = 14.48, *p* < 0.0001) were identified, whereas the overall group effect did not reach statistical significance (F(2, 21) = 2.686, *p* = 0.0915). Similarly, the immobility duration was significantly influenced by time (F(2.123, 44.57) = 15.90, *p* < 0.0001) and time × group interaction (F(4.245, 44.57) = 4.920, *p* = 0.0019), without a significant overall group effect (F(2, 21) = 1.990, *p* = 0.1616).

The open field behavioral parameters demonstrated moderate-to-large effect sizes across most analyses. The average locomotor speed showed large effects for time (partial η^2^ = 0.657), group (partial η^2^ = 0.509), and time × group interaction (partial η^2^ = 0.494). Similar findings were observed for locomotion duration (time: partial η^2^ = 0.573; group: partial η^2^ = 0.492; interaction: partial η^2^ = 0.561), time spent in the peripheral zone (time: partial η^2^ = 0.599; group: partial η^2^ = 0.571; interaction: partial η^2^ = 0.352), and time spent in the center zone (time: partial η^2^ = 0.577; group: partial η^2^ = 0.536; interaction: partial η^2^ = 0.381). The total locomotor distance was mainly influenced by time (partial η^2^ = 0.612) and time × group interaction (partial η^2^ = 0.580), while immobility duration demonstrated moderate effect sizes for time (partial η^2^ = 0.431) and interaction effects (partial η^2^ = 0.319).

The qualitative (Figure 2) and quantitative (Figure 3) analyses demonstrate that the onset of diabetes causes a progressive deterioration in locomotor activity and exploratory behavior, characterized by a reduced distance traveled, a decreased speed of movement, increased immobilization, and avoidance of the central area.

### 3.3. Electrophysiological Analysis

**Figure 4:**
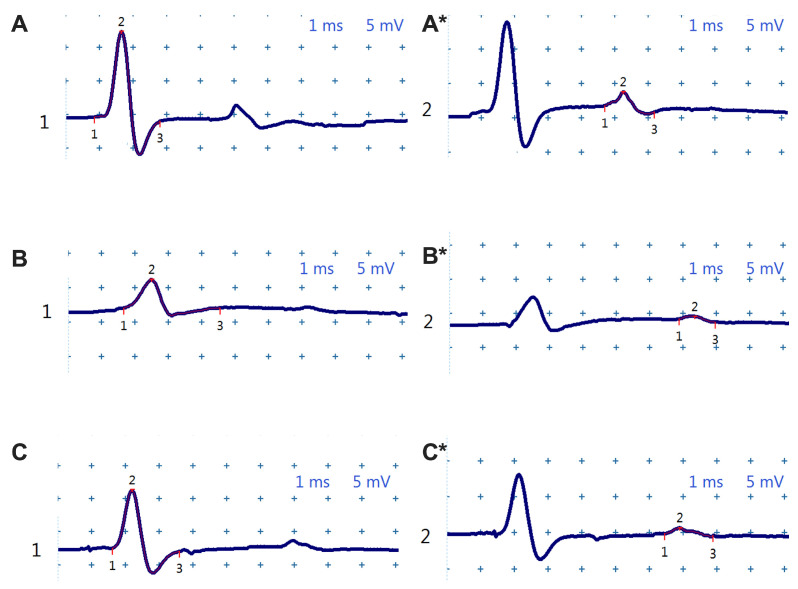
Representative images highlighting CMAP (points marked 1, 2, and 3) in non-diabetic sham mice (**A**), untreated diabetics (**B**), and diabetics treated with uridine (**C**). (*) Representative images with F-wave (points marked 1, 2, and 3) in non-diabetic sham mice (A*), untreated diabetics (B*), and diabetics treated with uridine (C*).

The electrophysiological analysis, presented in Figure 4, highlights progressive changes in nerve conduction in untreated diabetic mice, as well as the neuroprotective effects of chronic uridine administration. Compound motor action potential (CMAP) and F-wave recordings visually capture the degradation in neuromuscular transmission with the evolution of diabetes, while uridine treatment attenuates an important part of these alterations.

In healthy mice (sham group), CMAP (Figure 4A) shows large amplitudes and short durations, consistent with the optimal structural and functional integrity of the sciatic nerve. In contrast, in the untreated diabetic group (Figure 4B), the CMAP amplitude is visibly reduced, and the duration is prolonged, suggesting a mixed lesion, both axonal and demyelinating.

At the same time, the delineation of the F wave (marked with * in the figure) highlights delays in the proximal response, characteristic of early diabetic neuropathy. At 12 weeks, these changes become evident: the F wave is reduced in amplitude and significantly delayed, and the CMAP shows a constant decrease in amplitude.

In contrast, diabetic mice treated with uridine (Figure 4C) show CMAP and F wave tracings in between the sham and DM+ groups, with higher amplitudes and shorter latencies compared to untreated diabetic animals. This maintenance of the electrophysiological profile suggests a real neuroprotective effect on peripheral motor fibers.

**Figure 5:**
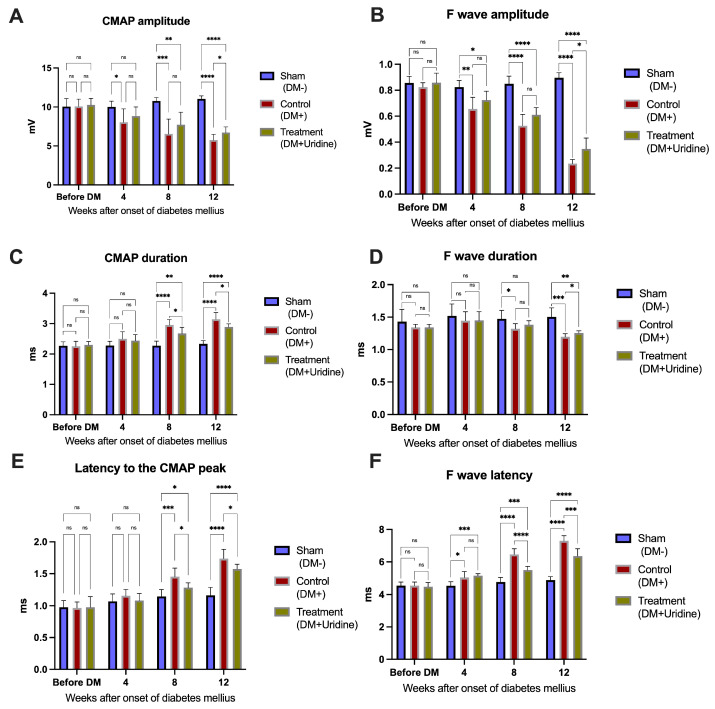
Electrophysiological parameters of the sciatic nerve at 0, 4, 8, and 12 weeks after the induction of diabetes. (**A**) CMAP amplitude; (**B**) F-wave amplitude; (**C**) CMAP duration; (**D**) F-wave duration; (**E**) F-wave latency; (**F**) latency to CMAP peak. Data are presented as the mean ± SD. Significance levels: * *p* < 0.05, ** *p* < 0.01, *** *p* < 0.001, **** *p* < 0.0001. ns, not significant.

The qualitative interpretation of these observations can be determined from the quantitative values presented in Figure 5, where the evolution of the electrophysiological parameters is analyzed comparatively before the induction of diabetes and then at 4, 8, and 12 weeks after streptozotocin administration.

The CMAP amplitude decreases significantly with the progression of diabetes: at 12 weeks, DM+ mice present a mean amplitude of approximately 5.75 ± 0.72 mV, significantly lower than in the sham group (11.03 ± 0.38 mV, *p* < 0.0001). In the uridine-treated group (DM+Uridine), the amplitude is significantly higher than in the DM+ group (*p* < 0.0001), remaining at a value close to that of the healthy group, which confirms the protective role of uridine on axonal integrity. Two-way repeated measures ANOVA demonstrated significant effects of time (F(2.328, 48.88) = 32.43, *p* < 0.0001), experimental group (F(2, 21) = 24.57, *p* < 0.0001), and time × group interaction (F(4.656, 48.88) = 18.12, *p* < 0.0001) on the CMAP amplitude.

The CMAP duration, an indicator of demyelination, progressively increases in the untreated diabetic group. At 8 weeks, the CMAP duration in DM+ mice is significantly longer compared to the sham group (*p* < 0.0001), and this trend is accentuated at 12 weeks. Uridine administration significantly reduces this prolongation at 8 weeks (*p* = 0.0016) and at 12 weeks (*p* < 0.0001), confirming an improvement in the integrity of the myelin sheath. Two-way repeated measures ANOVA revealed significant effects of time (F(2.589, 54.38) = 98.85, *p* < 0.0001), experimental group (F(2, 21) = 20.70, *p* < 0.0001), and time × group interaction (F(5.179, 54.38) = 24.01, *p* < 0.0001) on the CMAP duration.

The effects of diabetes on the proximal response are even more evident in the analysis of the F wave. The amplitude of the F wave decreases rapidly after the onset of diabetes, and at 12 weeks, the DM+ group shows markedly reduced values, approximately 0.23 ± 0.03 mV, compared to 0.89 ± 0.03 mV in the sham group (*p* < 0.0001). Mice treated with uridine maintain a significantly higher amplitude (0.34 ± 0.08 mV), superior to the DM+ group (*p* < 0.0001), suggesting functional protection of the proximal nerve segments. Repeated measures two-way ANOVA demonstrated significant main effects of time (F(2.575, 54.07) = 148.6, *p* < 0.0001) and group (F(2, 21) = 141.1, *p* < 0.0001), as well as a significant time × group interaction (F(5.149, 54.07) = 49.55, *p* < 0.0001) for the F-wave amplitude.

The duration of the F wave remains relatively stable up to 8 weeks, but at 12 weeks, the DM+ group shows a significant increase in duration (*p* = 0.0006). In the treated group, this increase is less pronounced (*p* = 0.0030), suggesting preservation of proximal conduction. Two-way repeated measures ANOVA showed that the F-wave duration was significantly influenced by time (F(2.207, 46.35) = 11.50, *p* < 0.0001), experimental group (F(2, 21) = 7.587, *p* = 0.0033), and the interaction between time and treatment (F(4.414, 46.35) = 3.132, *p* = 0.0199).

The latency of the F wave is one of the most sensitive indicators of diabetic neuropathy. Diabetes causes a rapid and severe increase in latency, reaching values of over 7.29 ± 0.31 ms at 12 weeks, compared to 4.89 ± 0.19 ms in the sham group (*p* < 0.0001). Uridine significantly reduces this delay, with treated mice presenting intermediate values (6.35 ± 0.45 ms), significantly lower than in DM+ (*p* < 0.0001), indicating a substantial improvement in nerve impulse propagation in proximal segments. Analysis using two-way repeated measures ANOVA identified significant effects of time (F(2.763, 58.02) = 211.3, *p* < 0.0001), treatment group (F(2, 21) = 79.75, *p* < 0.0001), and time × treatment interaction (F(5.526, 58.02) = 42.07, *p* < 0.0001) on the F-wave latency.

Finally, the latency to peak CMAP confirms the same pattern: at 12 weeks, diabetic mice present a significant increase in latency (*p* < 0.0001), while uridine treatment reduces this increase by over 70% (*p* < 0.0001). Statistical analysis using two-way repeated measures ANOVA revealed significant effects of time (F(2.700, 56.70) = 116.6, *p* < 0.0001), group (F(2, 21) = 21.46, *p* < 0.0001), and time × group interaction (F(5.400, 56.70) = 13.96, *p* < 0.0001) on the latency to the CMAP peak.

The electrophysiological parameters demonstrated predominantly large effect sizes. The CMAP amplitude showed strong effects for time (partial η^2^ = 0.607), group (partial η^2^ = 0.701), and time × group interaction (partial η^2^ = 0.633), while the CMAP duration exhibited similarly large effects (time: partial η^2^ = 0.825; group: partial η^2^ = 0.663; interaction: partial η^2^ = 0.696). The F-wave latency demonstrated particularly pronounced effect sizes (time: partial η^2^ = 0.910; group: partial η^2^ = 0.884; interaction: partial η^2^ = 0.800), whereas the F-wave amplitude also showed robust effects (time: partial η^2^ = 0.876; group: partial η^2^ = 0.931; interaction: partial η^2^ = 0.825). More moderate effects were observed for the F-wave duration (time: partial η^2^ = 0.354; group: partial η^2^ = 0.419; interaction: partial η^2^ = 0.230). The latency to the CMAP peak demonstrated large effect sizes for time (partial η^2^ = 0.847), group (partial η^2^ = 0.671), and interaction effects (partial η^2^ = 0.571).

Overall, the results presented in Figure 4 and Figure 5 clearly demonstrate that STZ-induced diabetes progressively impairs the sciatic nerve function, involving both distal (evidenced by CMAP changes) and proximal (evidenced by the F-wave) components. Chronic uridine administration significantly attenuates these changes, suggesting a consistent neuroprotective effect at the level of the motor axon, myelin sheath, and proximal nerve root circuits.

### 3.4. Histological Analysis

**Figure 6:**
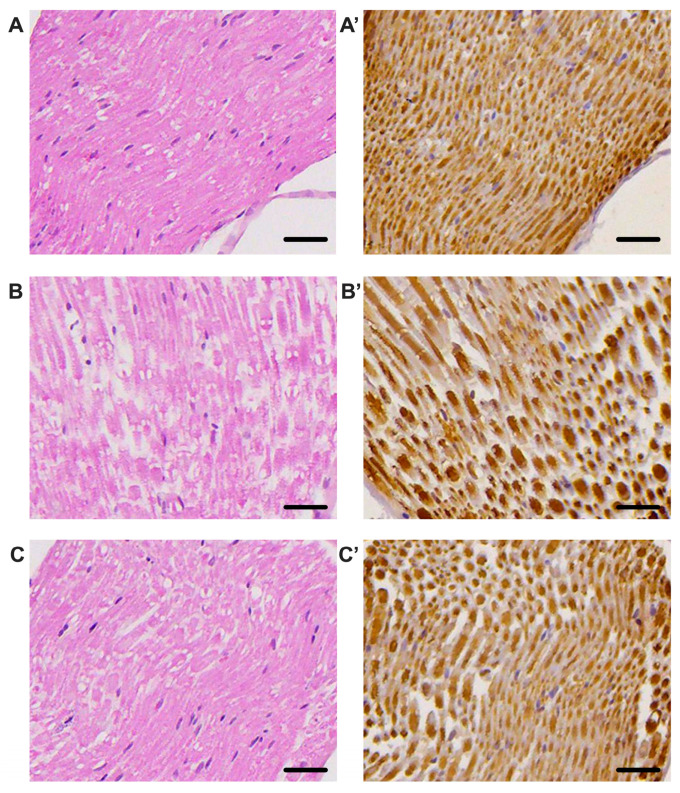
Histological and immunohistochemical aspects of the sciatic nerve in the experimental groups. (**A**–**C**) HE sections of the sciatic nerve in sham (**A**), untreated diabetic (**B**), and uridine-treated diabetic (**C**) mice. (**A′**–**C′**) Immunohistochemistry of PGP 9.5 in the same experimental groups. Scale bars represent 10 µm.

The histological analysis of the sciatic nerve, shown in Figure 6, reveals profound morphological changes associated with STZ-induced diabetes and demonstrates the protective effects of uridine treatment.

In hematoxylin–eosin (HE) staining, sections from healthy mice (Figure 6A) show a well-organized structure, with uniform nerve fibers and discrete endoneurial spaces. This regular architecture reflects the structural integrity of the sciatic nerve under physiological conditions. In contrast, nerve sections from untreated diabetic mice (Figure 6B) reveal obvious morphological changes: fibers appear thinner with variable contour; endoneurial spaces are widened, suggesting edema and axonal degeneration. These changes are characteristic of diabetic neuropathy and confirm the marked loss of structural integrity. Interestingly, HE sections from the uridine-treated group (Figure 6C) show a much closer architecture to normal than the untreated diabetic group. Fibers are better defined, and interfascicular spaces are reduced. Although they do not reach the appearance of the sham group, nerve sections from the DM+Uridine group show significant preservation of axonal structure, suggesting a neuroprotective effect.

**Figure 7:**
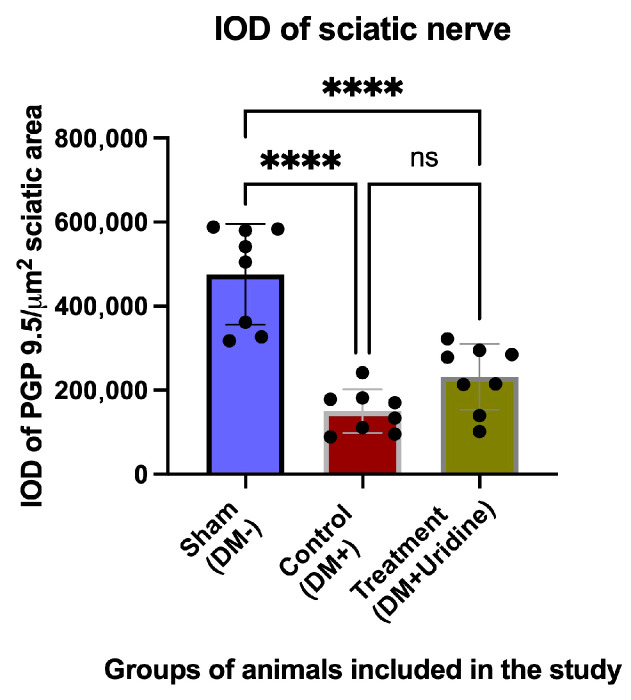
Integrated optical density (IOD) of the neuronal marker PGP 9.5 relative to the total area of the sciatic nerve section. Values are expressed as the mean ± SD. Significance levels: **** *p* < 0.0001. ns, not significant.

These morphological observations are supported by the immunohistochemistry for the pan-neuronal marker PGP 9.5, illustrated in Figure 6A′–C′. In the sham group (A′), PGP 9.5 shows a uniform and intense distribution, reflecting the integrity of the nerve fibers. In diabetic mice (B′), the PGP 9.5 signal is diminished, and the signal density is visibly reduced. In contrast, uridine treatment significantly increases the intensity of the PGP 9.5 signal (C′): the fibers are more numerous, their distribution is more uniform, and the axonal integrity seems partially restored. This suggests that uridine protects the nerve fibers, limiting the degeneration associated with diabetes. These qualitative observations are supported by the quantitative analysis of the integrated optical density (IOD), shown in Figure 7. The mean IOD value in the sham group is 475.793 ± 119.842, reflecting the increased density of intact fibers. In the untreated diabetic group, the IOD decreases drastically to 150.210 ± 52.099, which represents a significant reduction in nerve density (difference sham vs. DM+: 325.584, *p* < 0.0001). The difference between the DM+ group and the DM+Uridine group (approximately +81.174, *p* = 0.18) does not reach statistical significance in the direct analysis. Additional images of sciatic nerve sections stained with HE and PGP 9.5 are presented in Supplementary Figures S1–S12.

This observation suggests that uridine induces a partial improvement in nerve integrity. However, the increase in PGP 9.5 density observed in the histological images and the higher IOD values in the treated group indicate a real biological effect.

## 4. Discussion

The results of the present study demonstrate that chronic administration of uridine exerts neuroprotective effects in streptozotocin-induced diabetic neuropathy, favorably influencing the locomotor function, electrophysiological parameters, and histological aspects of the sciatic nerve. A central aspect is that the functional and structural benefits occur independently of glycemic control, as uridine did not modify the course of glycemia, body weight, or the HbA1c values compared to the untreated diabetic group, which supports the hypothesis that the mechanisms of action of uridine are predominantly neuroprotective and not metabolic.

Uridine may exert neuroprotective effects through multiple complementary mechanisms acting on neuronal metabolism, membrane integrity, oxidative stress, and nerve regeneration [26,27,28]. Experimental studies have shown that uridine promotes neurite outgrowth and neuronal branching, suggesting a direct trophic effect on peripheral nerve cells. In animal models of nerve injury, uridine administration reduced apoptotic and oxidative stress markers, including caspase-3, myeloperoxidase (MPO), and malondialdehyde (MDA), supporting its antiapoptotic and antioxidant properties [29]. In addition, uridine is a key precursor for the synthesis of cytidine-5′-diphosphocholine (CDP-choline), an essential intermediate in phospholipid biosynthesis, which is required for neuronal membrane repair, axonal growth, and myelin regeneration [27]. Uridine-mediated synthesis of membrane components such as phosphatidylcholine and phosphatidylinositol may further contribute to the preservation of peripheral nerve structure and function. Previous clinical studies in diabetic neuropathy have also demonstrated improvements in nerve conduction velocity, nerve fiber regeneration, myelin sheath integrity, and axonal thickness following chronic uridine administration [27]. Moreover, uridine may influence glucose metabolism through the formation of uridine diphosphate glucose, promoting glycogen synthesis and potentially reducing intracellular glucose accumulation and sorbitol-related neurotoxicity [27]. Collectively, these mechanisms may explain the functional and structural neuroprotective effects observed in diabetic peripheral neuropathy, independent of direct glycemic control.

Recent experimental data suggest that uridine may also exert important neuroprotective effects in the central nervous system through mitochondrial and metabolic mechanisms [16,22,28]. Uridine-derived phosphonucleotides, particularly UDP, can activate mitochondrial ATP-dependent potassium channels (mitoKATP), which are involved in tissue protection under conditions of oxidative stress and hypoxia. Activation of these channels has been associated with reduced oxidative damage, improved calcium homeostasis, and prevention of the opening of the mitochondrial calcium-dependent pore, thereby limiting neuronal injury and cell death [16]. In experimental neurodegenerative models, uridine administration reduced markers of oxidative stress and mitochondrial dysfunction, restored mitochondrial ultrastructure, and improved motor activity in a dose-dependent manner. In addition, uridine can improve cellular energy metabolism through multiple interconnected mechanisms [27]. By conversion to UTP and subsequently to UDP–glucose, uridine can support glycogen synthesis, and its degradation to uracil can increase acyl-CoA availability, favoring Krebs cycle activity and mitochondrial respiratory function [22]. UTP can also be converted to CTP, an essential substrate for phospholipid synthesis and membrane repair, including the maintenance and regeneration of myelin structures [22]. These mechanisms may contribute not only to peripheral nerve protection but also to beneficial effects on the central nervous system, possibly explaining the improvement in locomotor and anxiety behavior observed, independent from glycemic control [27].

### 4.1. Assessment of Locomotor Behavior

In the open field test, untreated diabetic mice showed significant reductions in the distance traveled, average speed, and time spent in the central zone, consistent with the motor impairment and increased anxiety-like behavior commonly observed in diabetic neuropathy. Uridine-treated animals showed consistent improvements in all these parameters, maintaining a trajectory pattern close to that of the sham group, even at 12 weeks.

These results suggest that uridine may modulate not only motor function but also anxiety-related behaviors, possibly through effects on peripheral nerve integrity or secondary central mechanisms. In animal studies, particularly in rat sciatic nerve injury models, systemic administration of uridine has been shown to significantly reduce apoptosis and oxidative stress in the injured nerve [27]. Other research on pyrimidine nucleotides—particularly combinations of cytidine and uridine—shows that these substances accelerate neuromuscular recovery after nerve injury and reduce pain transmission to the spinal cord [28].

Although the evidence comes mainly from animal models, the consistency of the results has attracted interest in clinical application [29,30,31,32,33].

In humans, however, uridine has rarely been studied as a monotherapy, being used mainly in combination with cytidine and B vitamins, especially vitamin B12, but sometimes also B1 or B6 [33,34,35,36,37,38,39,40]. In various studies in patients with painful peripheral neuropathy, diabetic neuropathy, or low back pain with a neuropathic component, these combinations have resulted in decreased pain intensity and improvement in sensory symptoms [36,37]. Compared to standard treatments or the simple administration of B vitamins, formulations that include uridine and cytidine have been shown to be more effective, including in randomized trials of radiculopathies and nerve compression syndromes [36,37].

Thus, for peripheral nerves, the scientific basis is solid: there is consistent preclinical evidence and clinical trials supporting the use of combinations of uridine, cytidine, and vitamin B12 in relieving neuropathic pain and supporting nerve regeneration [37]. However, because these substances are almost always administered together, it is difficult to determine the exact contribution of uridine.

There are also older data suggesting improvements in nerve conduction tests in patients with diabetic neuropathy, albeit in small groups [40]. In our study, the electrophysiological changes observed in the untreated diabetic group—decreased CMAP amplitude, prolonged CMAP duration, and increased F-wave latency—reflect the mixed axonal and demyelinating damage typical of diabetic polyneuropathy. Uridine treatment attenuated these changes by increasing the amplitudes and reducing the latencies of both the CMAP and F-wave. The concomitant protection of distal (reflected by CMAP) and proximal (reflected by F-wave) segments suggests an extended action of uridine along the entire motor axon and on the myelin sheaths. These results are consistent with the clinical and preclinical literature, which shows that uridine and its derivatives can support nerve fiber regeneration, improve conduction velocity, and reduce neuropathic symptoms [40].

### 4.2. Effects on Anxious Behavior

Regarding the central nervous system and anxiety, the data are more nuanced. There are animal studies that suggest a possible anxiolytic effect of uridine, mediated by several neurotransmitter systems—adrenergic, dopaminergic, histaminergic, and serotonergic—indicating a complex action at the brain level [41,42].

Other experiments highlight an antidepressant-like effect in rats, accentuated by the association with omega-3 fatty acids [41,42]. An increase in phospholipids, such as phosphatidylcholine, has also been observed in certain brain regions after uridine administration, a process that may improve synaptic function and neuronal plasticity.

In addition, some studies on uridine derivatives have shown influences on sleep, including potentiation of the sedative effect of benzodiazepines or changes in EEG activity suggestive of sleep induction. Certain plant extracts with sedative effects appear to act by increasing endogenous uridine levels in the prefrontal cortex [41,43].

However, in humans, the evidence is limited. There are only a few small exploratory studies of the utility of uridine in bipolar depression, and the results mainly focus on depressive symptoms rather than anxiety [43]. Currently, there are no randomized clinical trials that clearly demonstrate a direct anxiolytic effect of uridine in humans [42,43,44,45,46,47,48]. The observed benefits may be attributed to uridine’s role in membrane phospholipid biosynthesis, myelin regeneration, and mitochondrial function. In addition, the recent literature suggests possible anti-inflammatory and antioxidant effects [42,43,44,45,46,47,48]. Although the exact mechanisms were not investigated in this study, future research should explore the influence of uridine on oxidative stress, neuroinflammation, blood–brain barrier integrity, and the expression of proteins involved in regeneration.

### 4.3. Study Limitations

Although the study provides valuable clues regarding the beneficial effects of uridine, the results should be interpreted with caution. First, the relatively small sample size limits statistical power, especially for the histological parameters, where the variability is higher.

In addition, the use of a single experimental model—STZ-induced diabetic neuropathy, mainly representative of type 1 diabetes—restricts the generalizability of the conclusions. For a complete picture, testing in models that reproduce the characteristics of type 2 diabetes would be necessary.

At the same time, the absence of molecular assessments prevents a thorough understanding of the mechanisms by which uridine could modulate inflammatory, oxidative, or remyelination processes. Furthermore, although the functional, electrophysiological, and histological results appear concordant, the study does not provide correlation analyses that directly link structural changes to functional recovery.

Thus, although promising, the conclusions remain open to further investigation.

## 5. Conclusions

The findings of the present study demonstrate that chronic uridine administration exerts significant neuroprotective effects in streptozotocin-induced diabetic neuropathy through mechanisms that appear to be largely independent from glycemic control. Although uridine treatment did not modify the blood glucose levels, HbA1c, or diabetes-associated weight loss, it significantly improved locomotor and exploratory behavior, enhanced electrophysiological parameters of sciatic nerve function (including CMAP and F-wave measurements), and reduced structural nerve damage observed via histological evaluation.

Further experimental and clinical investigations are warranted to better elucidate the molecular mechanisms underlying these effects and to determine the translational relevance of uridine therapy in human diabetic neuropathy.

## Data Availability

The original contributions presented in this study are included in the article and its Supplementary Materials. Further inquiries can be directed to the corresponding author.

## Supplementary Materials

The following supporting information can be downloaded at: https://www.mdpi.com/article/10.3390/biom16050750/s1. Figure S1: HE section of the sciatic nerve in sham group, 20×. Figure S2: PGP 9.5 section of the sciatic nerve in sham group, 20×. Figure S3: HE section of the sciatic nerve in sham group, 20×. Figure S4: PGP 9.5 section of the sciatic nerve in sham group, 20×. Figure S5: HE section of the sciatic nerve in untreated diabetic group, 20×. Figure S6: PGP 9.5 section of the sciatic nerve in untreated diabetic group, 20×. Figure S7: HE section of the sciatic nerve in untreated diabetic group, 20×. Figure S8: PGP 9.5 section of the sciatic nerve in untreated diabetic group, 20×. Figure S9: HE section of the sciatic nerve in treated diabetic group, 20×. Figure S10: PGP 9.5 section of the sciatic nerve in treated diabetic group, 20×. Figure S11: HE section of the sciatic nerve in treated diabetic group, 20×. Figure S12: PGP 9.5 section of the sciatic nerve in treated diabetic group, 20×.

## Abbreviations

The following abbreviations are used in this manuscript:

CMAP	Compound motor action potential
DM	Diabetes mellitus
OF	Open field
IOD	Integrated Optical Density
PGP	Protein Gene Product 9.5
