# Supplementary material for: Uridine Improves Locomotor Activity and Sciatic Nerve Integrity in a Mouse Model of Diabetes Mellitus
Source: Biomolecules. 2026 May 20;16(5):750. doi: 10.3390/biom16050750 (PMC13205074; doi:10.3390/biom16050750)
Supplement: Supplementary file 1 [file biomolecules-16-00750-s001.zip › biomolecules-4287536-supplementary.pdf]

Supplementary Material

Histological and immunohistochemical aspects of the sciatic nerve in the experimental groups.

HE sections of the sciatic nerve in sham group 20X

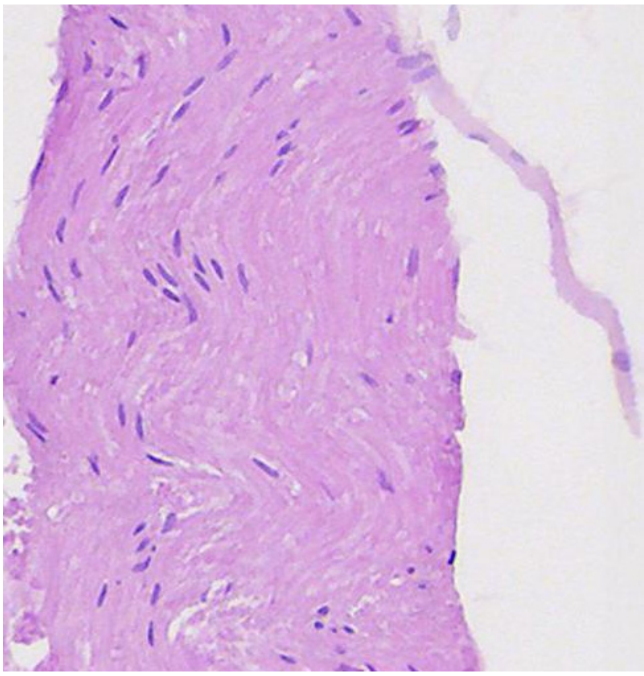

Figure S1, HE section of the sciatic nerve in sham group, 20X.

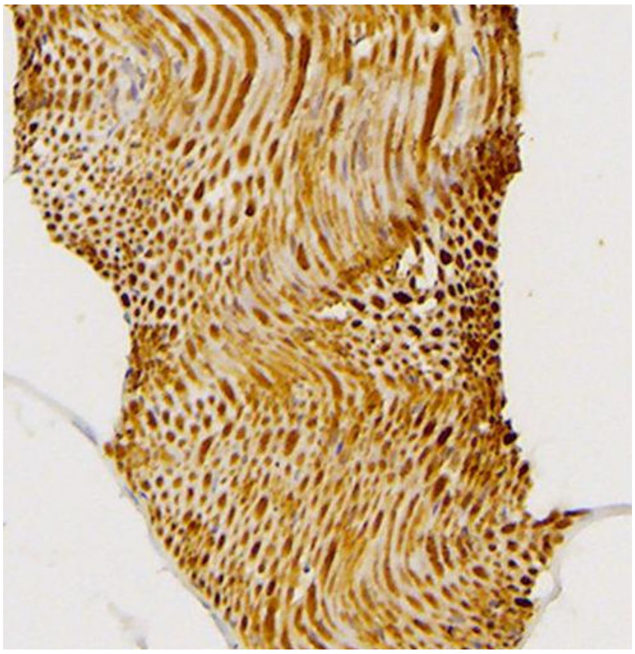

Figure S2. PGP 9.5 section of the sciatic nerve in sham group, 20X.

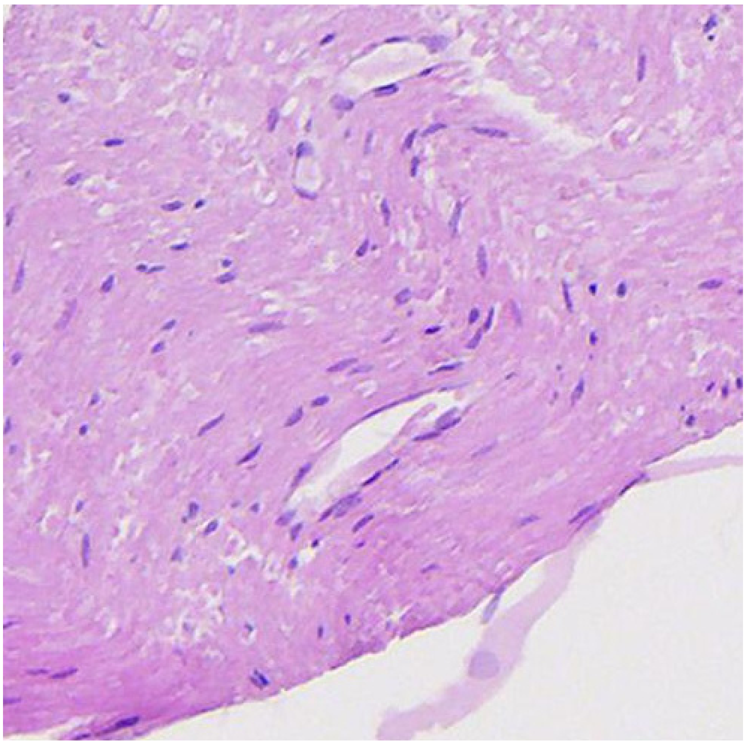

Figure S3. HE section of the sciatic nerve in sham group, 20X.

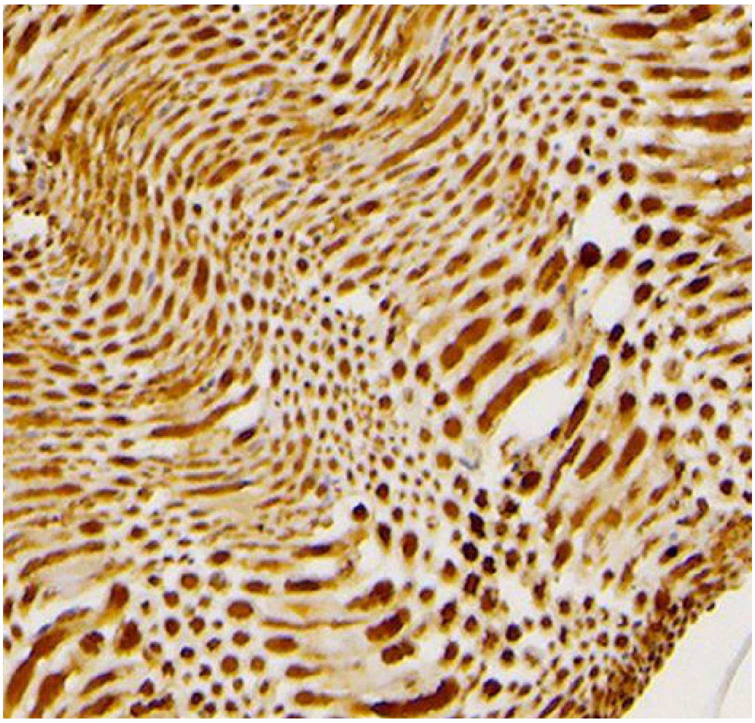

Figure S4. PGP 9.5 section of the sciatic nerve in sham group, 20X.

HE and PGP 9.5 sections of the sciatic nerve in untreated diabetic group 20X

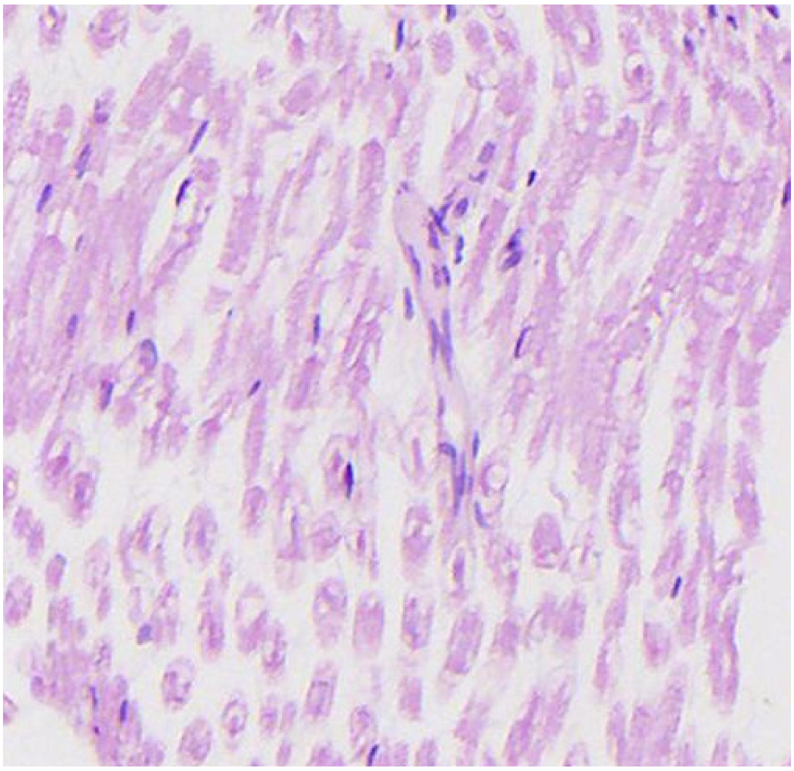

Figure S5. HE section of the sciatic nerve in untreated diabetic group, 20X.

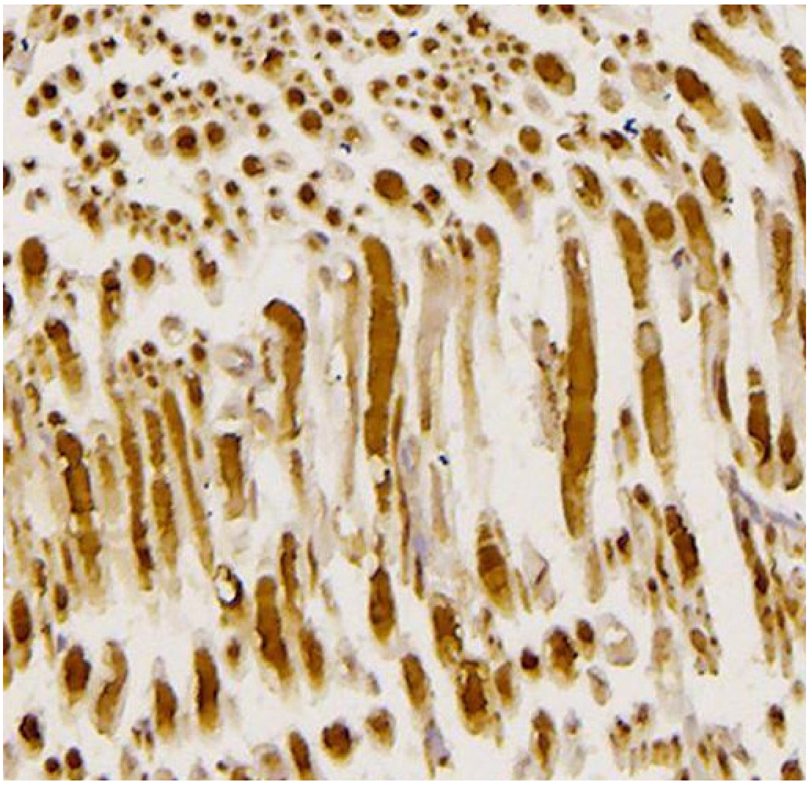

Figure S6. PGP 9.5 section of the sciatic nerve in untreated diabetic group, 20X.

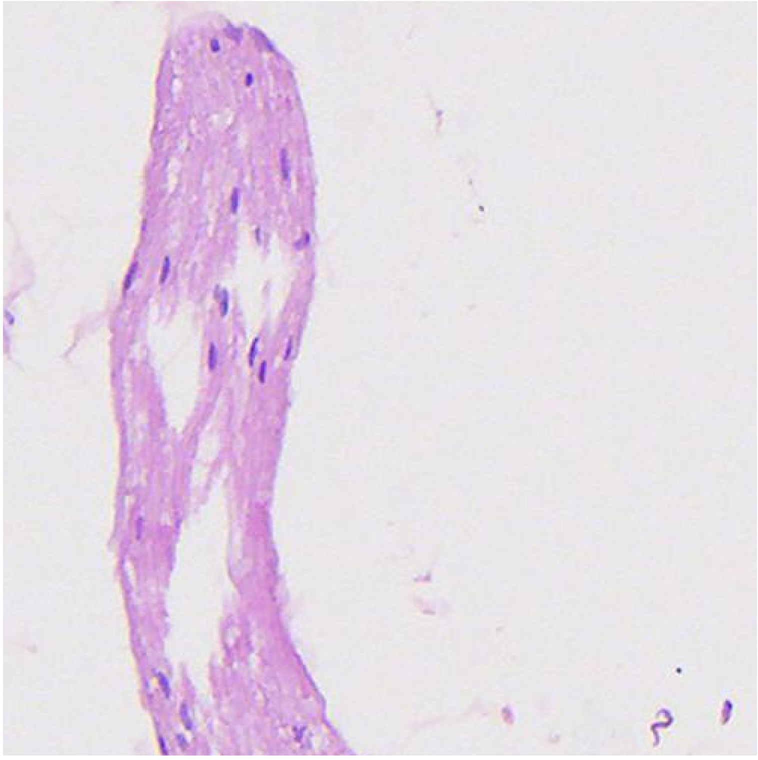

Figure S7. HE section of the sciatic nerve in untreated diabetic group, 20X.

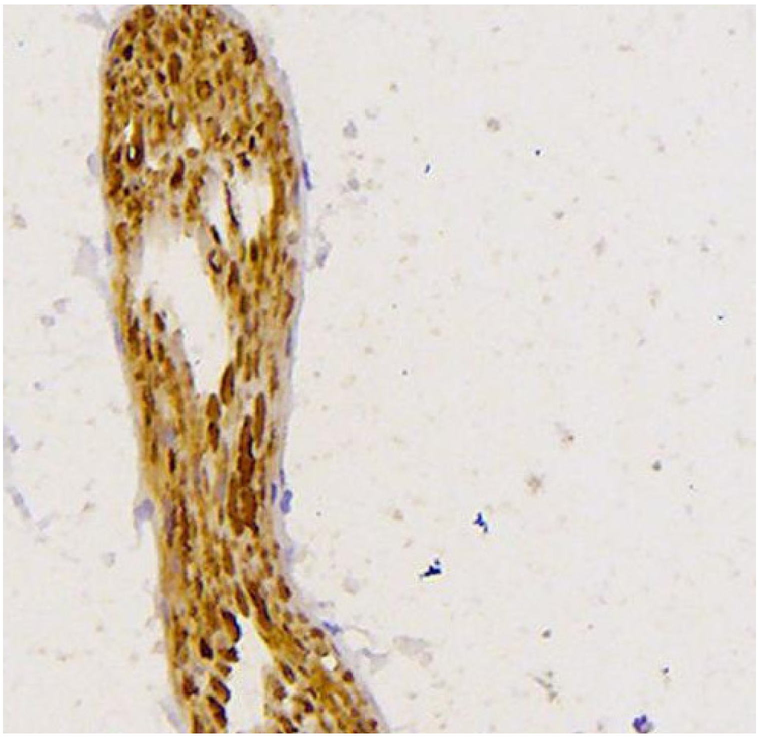

Figure S8. PGP 9.5 section of the sciatic nerve in untreated diabetic group, 20X.

HE sections of the sciatic nerve in uridine-treated diabetic group. 20X

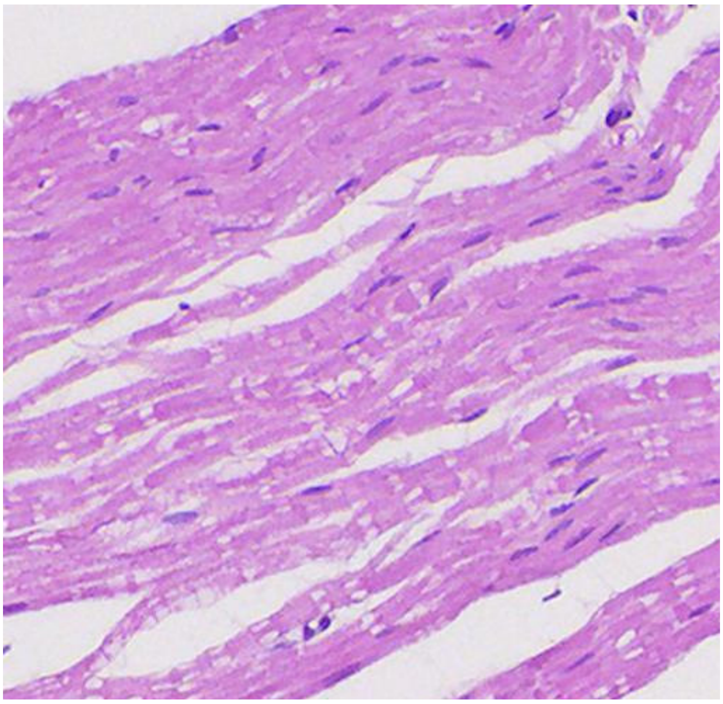

Figure S9. HE section of the sciatic nerve in treated diabetic group, 20X.

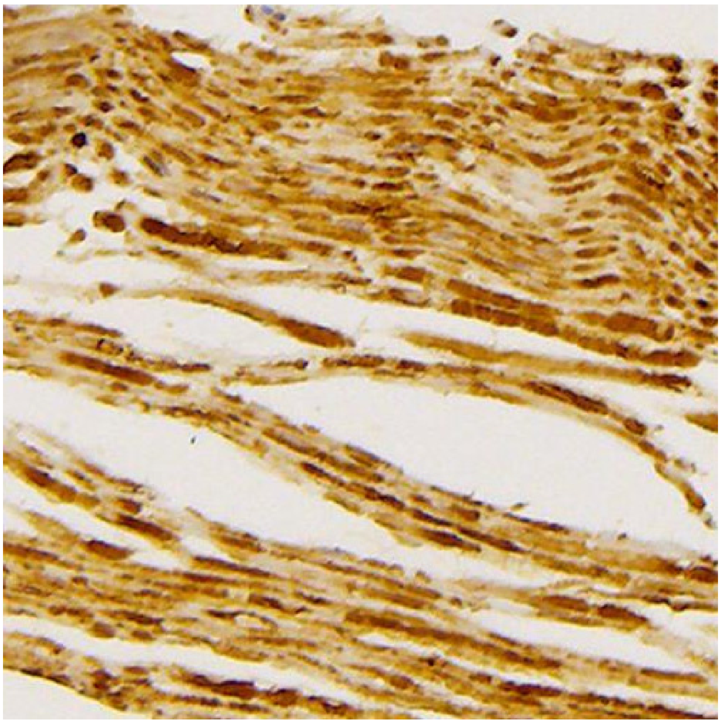

Figure S10. PGP 9.5 section of the sciatic nerve in treated diabetic group, 20X.

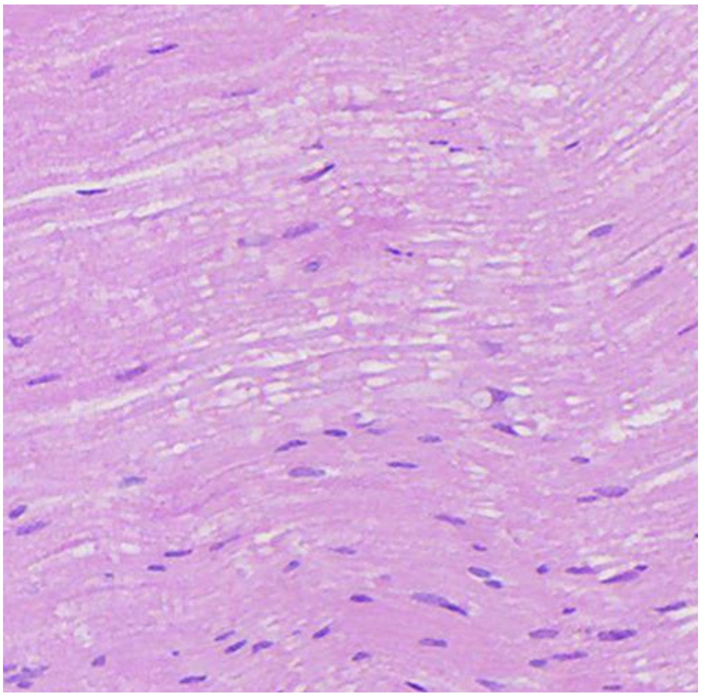

Figure S11. HE section of the sciatic nerve in treated diabetic group, 20X.

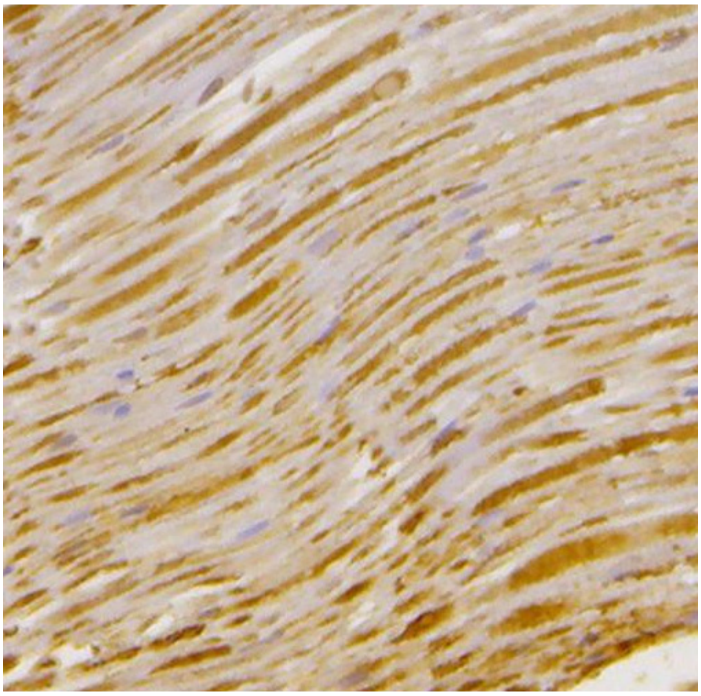

Figure S12. PGP 9.5 section of the sciatic nerve in treated diabetic group, 20X.
